# Elemental Composition of Above and Belowground Mangrove Tissue and Sediment in Managed and Unmanaged Compartments of the Matang Mangrove Forest Reserve

**DOI:** 10.3390/plants11212916

**Published:** 2022-10-29

**Authors:** Waseem Razzaq Khan, Mohammed Othman Aljahdali

**Affiliations:** 1 Department of Forestry Science, Faculty of Agricultural and Forestry Sciences, Universiti Putra Malaysia Kampus, Bintulu 97008, Malaysia; 2 Department of Biological Sciences, Faculty of Science, King Abdulaziz University, P.O. Box 80203, Jeddah 21589, Saudi Arabia

**Keywords:** *Rhizophora apiculata*, MMFR, Virgin Jungle Reserve (VJR), compartments, elemental pattern, stable isotopes

## Abstract

Mangrove productivity depends on the storage of nutrients and elements. Elemental concentrations were examined in leaves, roots, and sediments for three age stands (15, 25 years, and VJR) of *Rhizophora apiculata* in the Matang Mangrove Forest Reserve (MMFR). Six compartments with two compartments each for each age group were used to analyze sixteen elements. Four types of elemental patterns were examined with decreasing order during analysis: (1) Cd < Cu < Pb < Zn < Mg < Mn < Fe < K < Na < Ca and P% < S% < N% < C% in leaves, (2) Cd < Pb < Cu < Zn < Mg < Mn < Fe < K < Na < Ca and P% < S% < N% < C% in roots, (3) Cd < Pb < Cu < Zn < Mg < Mn < K < Fe < Na < Ca and P% < N% < S% < C% in sediment samples and (4) Cd_(S)_ < Pb_(S)_ < Cu_(S)_ < Zn_(S)_ < Mg_(S)_ < Mn_(L)_ < K_(L)_ < Fe_(S)_ < Na_(R)_ < Ca_(R)_ and P%_(S)_ < S%_(S)_ < N%_(L)_ < C%_(R)_ collectively for all samples. Evidence that elements do not store primarily in above-ground biomass can be found in the observation that elements are stored more in sediment and roots. The outcome of the present study shows that the rate of increase of elements in trees (leaves and roots) was less as compared to sediments, where the elemental concentration increased considerably with time. Elemental concentration comparison within three age classes showed that C, N, and S were significantly different in all three types of samples. The δ15N ratios showed positive values in all six compartments which supported the concept that the δ15N ratio could not be observed in N concentration in this study. The δ13C values showed more negative values in all six compartments which represented less salinity and a freshwater intake. The S, P, and heavy metals concentrations were high. The concentrations of Cd, P, N, C, and S in the sediment influenced variations in four compartments in accordance with the three mangrove age groups. The results of this study can be utilized to create management plans for MMFR and conduct risk assessments of the elements’ concentration in sediment.

## 1. Introduction

Mangrove ecosystems are highly productive in terms of storing and fixing high amounts of carbon [1,2,3]. Although mangroves are rich in carbon, there is a contradiction found in studies that some mangroves are poor in nutrient accumulation. Often mangrove soil is deficient in nutrients [4] but nutrient availability varies within and between mangrove forests [5]. According to Ukpong, nutrients exhibited a major component that could affect vegetation performance in Africa [6]. In Florida, the mangrove stand of *Rhizophora mangle,* which had more nutrients and freshwater input, was highly productive relative to mangrove species in other areas [7]. Furthermore, nutrient limitation is a key factor in causing productivity decline [8,9].

Mangroves can trap sediments that cause sedimentation. The high sedimentation rate provides a favorable condition for the deposition of elements. Further, these elements are absorbed by the roots and dispersed to the mangrove tree parts. In this scenario, mangroves are considered an indicator of metal pollution because they can accumulate metals and have survival tolerance [10]. The pattern of elemental use and storage in a forest ecosystem depends on many factors such as forest age, species composition, and soil fertility [11]. In a terrestrial forest ecosystem, productivity decline with forest age has been well documented. On the other hand, for mangroves, the use of nutrients with forest age is not well described. In Southeast Asia, *Rhizophora apiculata* forest stand canopy increases from young seedlings to approximately 20 years of age, after which growth is not uniform until the age of 65 years [12]. This also indicates that upon reaching the rotation stage, the canopy production of mangrove species declines at a later stage compared to other trees. In Thailand, nutrient cycling was examined in three age stands of *Rhizophora apiculata*: 3, 5, and 25 years. Results showed that with increasing age of the mangrove stand, nitrogen (N) percentage in submerged soil decreased, in contrast to increment in nutrient accumulation in mangrove trees [11,13,14]. This study examines nutrient use and storage in different ages of *Rhizophora apiculata* forest stand, which is an abundant specie along the coast of Southeast Asia and Australia.

Mangrove ecosystems are increasingly being affected by anthropogenic activities, which have contaminated the pristine environment conditions with pollutants including hazardous metals [15,16,17]. Metals and other elements are easily absorbed into coastal ecosystems, where they settle in benthic sediment and have the propensity to remain and build up in biological systems, causing physiological alterations [18]. Anthropogenic activities causing pollution, habitat degradation, and overexploitation of natural resources have intensive and negative effects on biodiversity and ecosystem services [19]. Anthropogenic stressors in coastal ecosystems causing environmental degradation, such as high concentrations of toxic metals from the urban environment, can accumulate in benthic sediment [20] However, the identification of sources of pollution is achieved using benthic sediments to evaluate pathways of distribution and detect sinks of pollutants in the aquatic ecosystem [17,19]. This is achieved as a result of the vital role of benthic sediment in the dynamics of pollutants [21]. 

The measurements of elemental concentration in mangroves and the identification of natural and anthropogenic contributions are important in assessing the quality of the mangrove ecosystem quality and restoration practice, and for providing valuable knowledge for the assessment of environmental health risks [21,22]. In addition, the assessment of the distributions and concentration of elements plays a crucial role in mangrove risk assessment and enables the restoration of coastal ecosystems [23,24]. Several studies have demonstrated that more than 90% of elements including metals are absorbed by sediments in suspended form, thus leading to accumulation in coastal environments [22]. 

The objective of the current study is to compare elemental concentrations present in the sampled compartments of the Matang Mangrove Forest Reserve (MMFR). This was carried out as follows: elemental concentrations were estimated in sediment, leaf and root samples of *Rhizophora apiculata* species aged 15 and 25 years, and Virgin Jungle Reserve (VJR). In addition, elemental concentrations were also compared between individual compartments for the 15-year age group, the 25-year age group, and VJR to determine whether any differences exist due to the location of the compartment. The stable isotopes tool was also used to identify the significant differences in elemental concentrations between sediment, leaf, and root samples because of environmental conditions. Stable isotope analysis is a commonly used technique to carry out various types of environmental assessment and monitoring, which gives an overview of biogeochemical processes over time. For instance, changes in stable isotopic compositions of 13C/12C and 15N/14N can help give insights into pathways and cycling of carbon and nitrogen [25]. Stable isotopes have also been used to study mangrove nutrient uptake [26] and mangrove freshwater use [27,28]. 

## 2. Results

### 2.1. Composition of Elements in Mangrove Leaves, Roots, and Sediments

A total of sixteen elements were analyzed in all six compartments (18, 31, 42, 71, 74 and 55) for *R. apiculata*. In leaves, the mean elemental concentration ranged from 3.57‰ to 8.67‰ for δ15N, −31.43‰ to −27.90‰ for δ13C, 0.006 to 0.0135 for Cd, 0.052 to 0.117 for Cu, 3.57 to 7.11 for Fe, 27.17 to 31.04 for K, 1.65 to 1.85 for Mg, 2.03 to 5.36 for Mn, 0.046 to 0.143 for Pb, 0.382 to 0.757 for Zn, 3881.06 to 4946.80 for Na, 11237.73 to 11318 for Ca, 34.25% to 41.74% for C, 0.970% to 12.01% for N, 0.222% to 0.328% for P and 0.287% to 0.419% for S. Cd < Cu < Pb < Zn < Mg < Mn < Fe < K < Na < Ca and P% < S% < N% < C% order was recorded according to the mean values of elements. Contents of C, Mn, Cd, Zn, Na, S, and Cu were found significant and placed in separate groups (Table 1).

In root samples of *R. apiculata*, mean values of elements ranged from 4.47‰ to 9.86‰ for δ15N, −28.40‰ to −20.64‰ for δ13C, 0.007 to 0.009 for Cd, 0.0602 to 0.115 for Cu, 4.91 to 7.69 for Fe, 23.92 to 28.07 for K, 1.76 to 1.83 for Mg, 0.582 to 2.069 for Mn, 0.067 to 0.087 for Pb, 0.412 to 0.526 for Zn, 5105.06 to 5798.6 for Na, 10765.68 to 11329.4 for Ca, 37.03% to 40.89% for C, 0.392% to 0.551% for N, 0.200% to 0.268% for P and 0.211% to 0.535% S. Decreasing order of Cd < Pb < Cu < Zn < Mg < Mn < Fe < K < Na < Ca and P% < S% < N% < C% were recorded in root samples. δ15N, δ13C, Ca, Na, C, and S showed significant results in all 16 variables and were placed in separate groups as shown in Table 2.

In sediment samples from all six compartments, elemental concentration mean values ranged from 2.66‰ to 6.67‰ for δ15N, −31.27‰ to −28.82‰ for δ13C,0.015 to 0.017 for Cd, 0.041 to 0.55 for Cu, 36.25 to 46.32 for Fe, 22.08 to 28.93 for K,1.65 to 1.96 for Mg, 0.882 to 2.16 for Mn,0.185 to 0.283 for Pb, 0.5 to 0.998 for Zn, 4851.66 to 5156.68 for Na, 9448.2 to 10817.86 for Ca, 7.27% to 10.84% for C,0.319% to 0.477% for N, 0.201% to 0.380% for P and 0.542% to 1.70% for S. A decreasing order of Cd < Pb < Cu < Zn < Mg < Mn < K < Fe < Na < Ca and P% < N% < S% < C% was observed. A comparison of sediment nutrients within all six compartments showed that δ15N, δ13C, Na, P, Cu, K, Mn, Zn, C, N, and S were significantly different (Table 3). A collectively decreasing order of Cd_(S)_ < Pb_(S)_ < Cu_(S)_ < Zn_(S)_ < Mg_(S)_ < Mn_(L)_ < K_(L)_ < Fe_(S)_ < Na_(R)_ < Ca_(R)_ and P%_(S)_ < S%_(S)_ < N%_(L)_ < C%_(R)_ in leaves, root, and sediment samples for all six compartments was observed and the highest concentration of δ15N and δ13C was observed in root samples.

### 2.2. Multivariate Analysis

**Figure 1:**
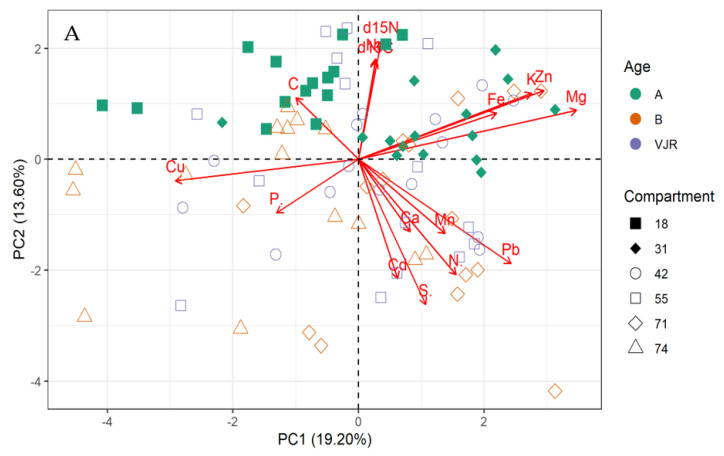

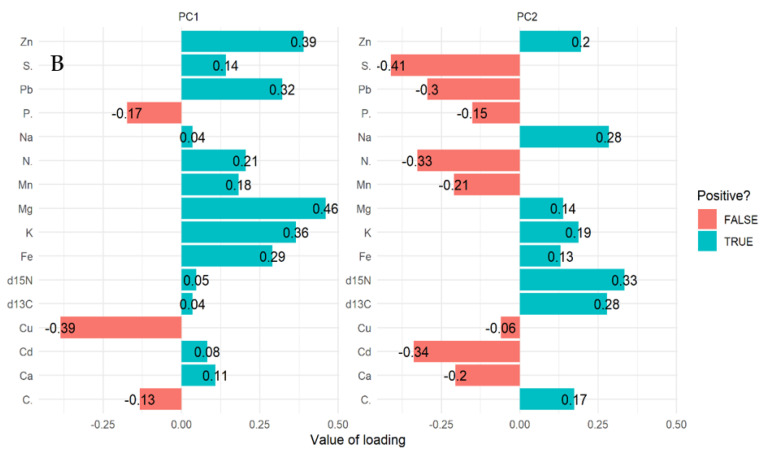
Relationship between elements in *Rhizophora apiculata* leaves, compartment, and compartment ages in the Matang Mangrove Forest Reserve, as determined by principal component analysis biplot (**A**) and loadings (**B**).

The multivariate principal component analysis (PCA) biplot for mangrove leaves revealed the influence of all the elements except Cu, C, and S on the variation of compartments (Figure 1A,B). According to this relationship, δ13C, δ15N C, Fe, K, Zn, Mg, and Na influences the differences between compartment 18 and 31 for *Rhizophora apiculata* species aged 15 from other compartments and ages (Figure 1A). However, Cu and P influence variation in all the compartments except 31 and 18 based on mangrove ages 25 and VJR. Relationships based on the compartment revealed compartment 18 to be more influenced by C, compartment 31 by Fe, K, Zn and Mg, Cu and P, while Pb, N, Cd, Ca, S and Mn by compartments 42, 55, 71 and 74. This relationship revealed by the PCA was based on component 1 (19.20%) and component 2 (13.60%) accounting for a total variation of 32.80% (Figure 1A).

**Figure 2:**
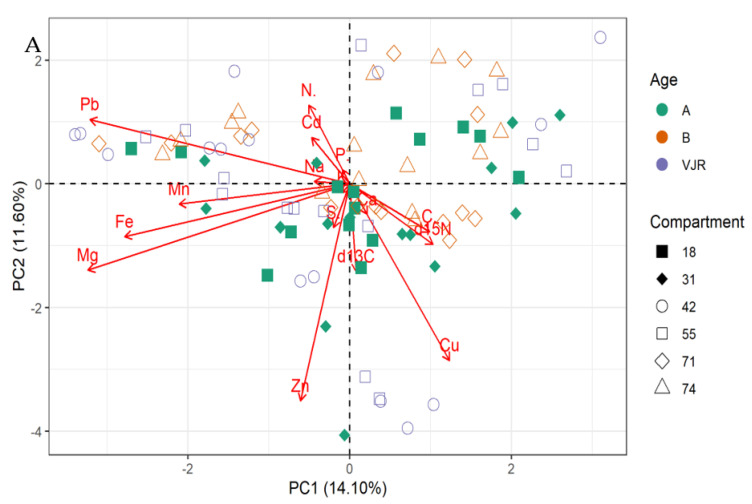

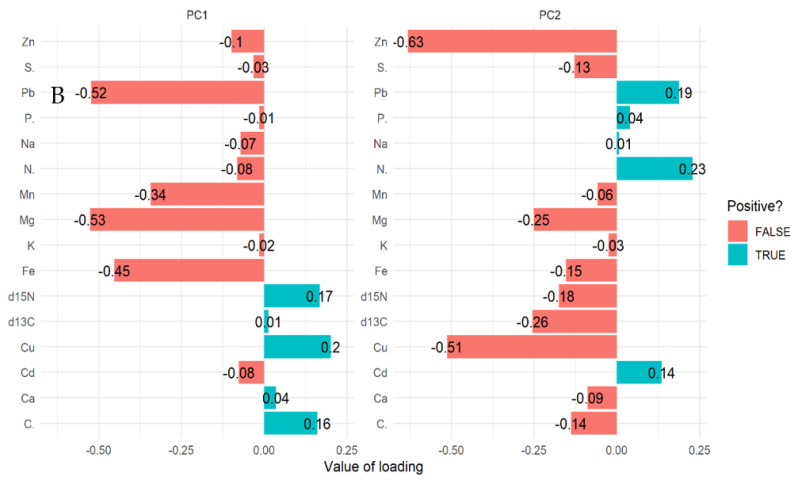
Relationship between elements in Rhizophora apiculata roots, compartment, and compartment ages in the Matang Mangrove Forest Reserve, as determined by principal component analysis biplot (**A**) and loadings (**B**).

The PCA biplot for mangrove roots revealed the influence of all the elements on the variation of compartments (Figure 2A,B). In line with this relationship δ13C, δ15N and all the elements influence the variations in all the compartments for *Rhizophora apiculata* species aged 15, 25, and VJR (Figure 2A). However, δ13C, δ15N, C, Ca, and Cu were negatively correlated with other elements (Figure 2A,B). This relationship revealed by the PCA was based on component 1 (14.10%) and component 2 (11.60%) accounting for a total variation of 25.70% (Figure 2A).

**Figure 3:**
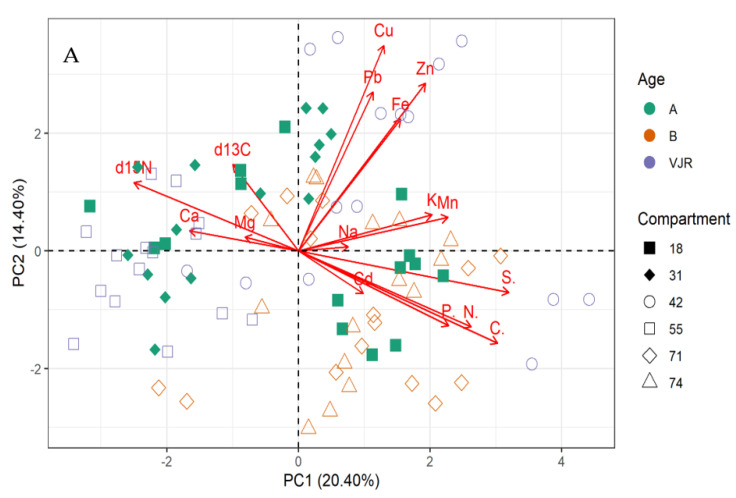

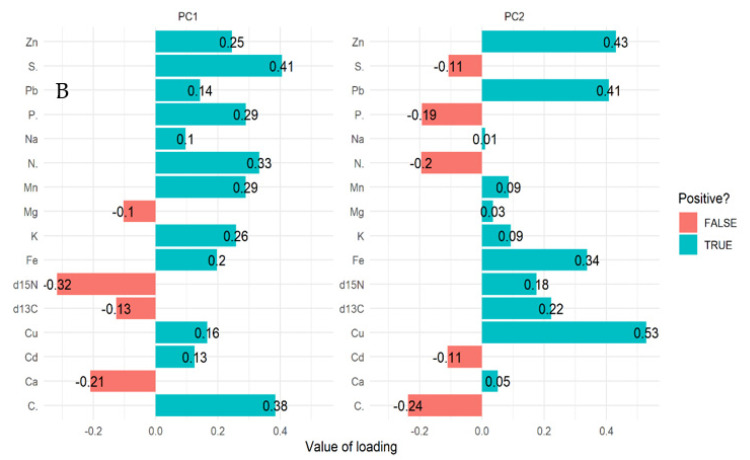
Relationship between elements in sediment, compartments, and compartment ages in the Matang Mangrove Forest Reserve as determined by principal component analysis biplot (**A**) and loadings (**B**).

For mangrove sediment, the PCA revealed component 1 (20.40%) and component 2 (14.40%) account for 34.80% of the total variation. The PCA biplot for mangrove sediment revealed the influence of δ13C, δ15N, Ca, and Mg on compartments 18, 31, 55, 71, and 74 (Figure 3A,B). However, Pb, Cu, Zn, Fe, Na, K, and Mn influenced compartments 18, 31, 42, 71, and 74. In addition, compartments 18, 42, 71, and 74 were influenced by Cd, P, N, C, and S based on the three mangrove age groups (15 years, 25 years, and VJR) (Figure 3A,B). However, δ13C, δ15N, Ca, and Mg were negatively correlated with Pb, Cu, Zn, Fe, Na, K, Mn, Cd, P, N, C, and S (Figure 3A).

### 2.3. Elemental Pattern and Mangrove Age

**Figure 4:**
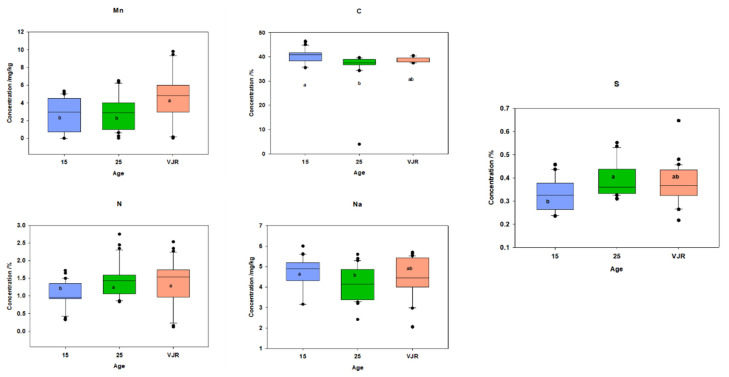
Elemental concentration in leaves (mean was taken for the same age compartments) in boxplot representation with the median, interquartile range, and outliers (black dots). Small letters (a, b) show different significance groups where the significance level was set as *p* < 0.05.

**Figure 5:**
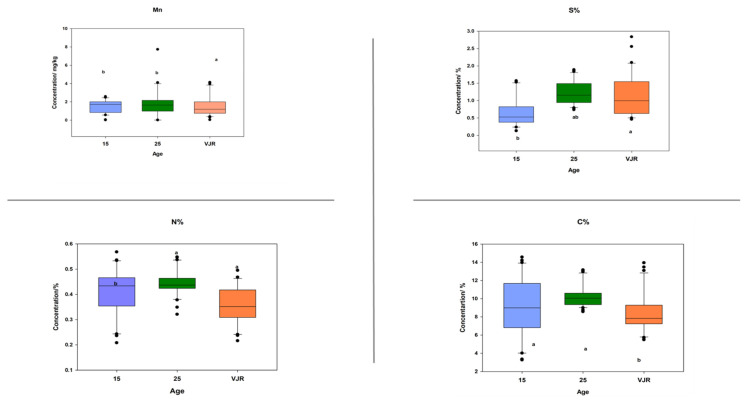
Elemental concentration in roots (the mean was taken for the same age compartments) in boxplot representation with the median, interquartile range, and outliers (black dots). Small letters (a, b) show different significance groups where the significance level was set as *p* < 0.05.

**Figure 6:**
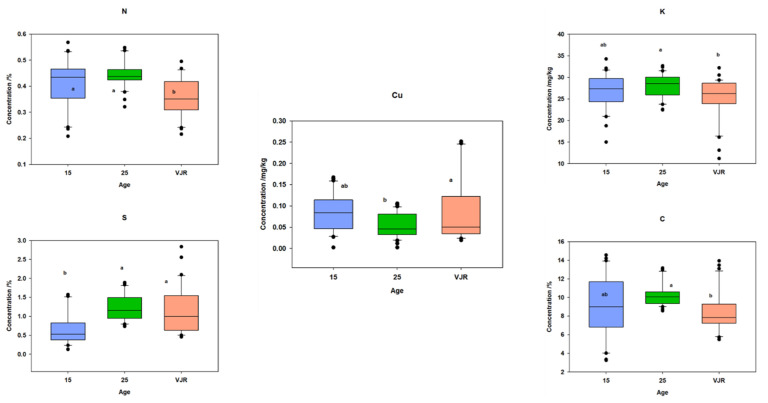
Elemental concentration in sediments (the mean was taken for the same age compartments) in boxplot representation with the median, interquartile range, and outliers (black dots). Small letters (a, b) show different significance groups where the significance level was set as *p* < 0.05.

To observe the elemental pattern with increasing age, three age classes of 15 years, 25 years, and VJR were established in the box plot. This was completed by taking the sample mean values of compartments 18 and 31 for the 15-year age class, 71 and 74 for the 25-year age class, and 42 and 55 for the VJR. This analysis presented a significant difference among certain elements in different age groups in all three categories: leaves, roots, and sediments. In leaf samples, Mn, Na, C, N, and S showed significant differences in the 15-year, 25-year, and VJR classes (Figure 4). Moreover, in root samples, Mn, C, N, and S showed significant differences within the three age classes (Figure 5). Lastly in the sediment samples, Cu, K, C, N, and S showed significant differences in the three age classes (Figure 6). Interestingly, in all three types of samples C, N, and S showed substantial differences which shows that concentrations of these elements change with time continuously. This analysis is also in contrast to the analysis of the results presented earlier where averages of each class were taken rather than the means of the same age group. The earlier analysis showed no difference among different age classes.

The variations between compartments 18 and 31 for *Rhizophora apiculata* species aged 15 from other compartments and ages are influenced by 13C, 15N C, Fe, K, Zn, Mg, and Na based on 32.80% total variation.

The δ13C, δ15N, and all the elements influence the variations in all the compartments for *Rhizophora apiculata* species aged 15, 25, and VJR based on 25.70 % total variation.

The δ13C, δ15N, Ca, and Mg influenced variation in compartments 18, 31, 55, 71, and 74, and Pb, Cu, Zn, Fe, Na, K, and Mn influenced compartments 18, 31, 42, 71, and 74 based on 34.80% total variation. 

## 3. Discussion

The storage of nutrients and elements in different concentrations determines the productivity of the mangrove’s ecosystem. Usually, mangroves are poor in stocking nutrients in the soil [4]. However, this paper finds that the sediments store more elements compared to the root and leaves (living biomass). Furthermore, this paper states that certain elemental compositions of the samples from leaves, roots, and sediments vary considerably in all three age groups. A similar idea is suggested in [12] which states that forest age is one of the many factors that determine the elemental storage in a forest ecosystem. This was also supported by the influence of sediments Cd, P, N, C, and S concentrations on variations in compartments 18, 42, 71, and 74, in accordance with the three mangrove age groups revealed by the PCA with a total variation of 34.80%. The current study also observed that the concentration of most elements in leaves and roots increased with age. The pool size of all the elements in sediment increased with age except C, N, Mn, and Pb. In the leaves and roots section, Cu. Cu, Cd, Pb, Mn, Zn, C, P, and S concentrations increased in the living biomass of trees with age. Nevertheless, some data patterns in other forest ecosystems revealed declining elemental concentrations with age [13]. The relation between age and elemental concentration is highly complex because of the interactions of climate, nutrient use, leaching, and soil fertility [29]. Alongi et al. [12] also argue that age-wise, *R. apiculata* in Southeast Asia shows a growth trend to 20 years of age thereafter, presenting an inconsistent trend up to the age of 65 [12]. This could also form the reason for δ13C, δ15N C, Fe, K, Zn, Mg, and Na influencing the differences between compartments 18 and 31 for *Rhizophora apiculata* species aged 15 from other compartments and ages.

Results from other studies presented a decline in nitrogen (N) percentage in the soil in three age stands of 3, 5, and 25 years. However, this nitrogen (N) increased or accumulated in the mangrove tree [11,13,14]. In the present study, most of the elements were accumulated in roots and sediment. The accumulation of elements in soil or subsurface soil was due to the slow decomposition of roots. Below-ground mud soil acts as a storage unit for elements [24]. For terrestrial forests, the storage of nutrients or elements is mainly concentrated on floor litter [25]. On the other hand, for mangroves, subsurface soil is the main storage compartment because tidal movement and crab foraging inhibit the storage of nutrients on surface soil [26]. Resultantly dead roots act as storage units for nutrients and can serve as prime sites for carbon sequestration. Soil element availability and storage are dependent on geochemical processes occurring in mangrove soil. Some other elements found in higher concentrations in the sediments were S and Fe. Roots prevent the transport of toxic materials such as sulfides by accumulating them [27]. Similar to the study in Swai Bay forests in Thailand, our results showed that the pattern of elements stored in sediment was high. Similar observations were made in *Avicennia marina* and *Rhizophora stylosa* mangrove forests, where 95% of all the elements were stored in soil [12,13].

The high content of nitrogen (N) in leaves sampled from compartment 31 indicates the influence of anthropogenic activities such as agricultural activities in the catchment of the compartment [30]. Other causes could include increased N cycling associated directly with anthropogenic impacts [31]. Agricultural waste is a product of anthropogenic activities and could contribute to an increase in N values. [32]. A possible influence of anthropogenic activities in mangrove ecosystems such as agricultural waste has been reported in the Matang mangrove ecosystem in Malaysia [1], New Zealand [33], and other impacted sites located in the central Red Sea [31]. In addition, other studies have also reported a high concentration of N in mangrove leaves compared to other mangrove tissues and sediment [34,35]. A possible presence of younger leaves in samples from compartment 31 could also form a part of the reason for the high N concentration recorded [34].

As for the trend-wise comparison, a study was conducted in 20-year-old aged compartments of MMFR [10]. Roots, stem disks, barks, twigs, and leaf samples were analyzed to find out the concentration of elements. The decreasing trend of Ca > Na > K > Mg > Mn > Al > Fe > Zn > Cu > Pb > Cd were stated. The present research showed a similar trend where Ca > Na > C > K > N > Fe > Mn > Mg > Zn > S > P > Pb > Cu > Cd was observed in leaves and root samples. Regarding the presence of elements, the moderate concentration of Mg, K, Ca, Na and C in leaves were found in studies that analyzed mangroves and tropical trees [13,36,37]. These studies also reported high concentrations of Cu, Cd, Pb, Mn, and Zn which can be interpreted as heavy metal pollution due to human activities [38]. Additionally, *Rhizophora* species gather Ca in leaves but keep K and Na at mid-level [16,17]. Furthermore, according to our research, the N and P percentage was different as compared to other mangrove studies [14,39]. This study does not support the findings of past studies that mangrove forest accumulates additional nutrients in living biomass compared to soil.

Within same-age compartments, elemental concentration was more varied between 18 and 31 (15 years) compared to variation between 71 and 74 (25 years). This could be due to widespread pollution in Kuala Sepetang where 15-year-old compartments are located [40]. This deviation might be caused by a change in situ, anthropogenic effects. Virgin Jungle Reserve compartments (VJR), which were of unknown age, exhibited similar behavior except for sulfur concentration. VJR 42 is in Kuala Sepetang, where high concentrations of sulfur might be due to pollution. Mg, K, Ca, Na, and P concentrations in living biomass and sediment were almost the same in all the compartments. Analysis of P concentration showed that the area was not deficient in P. Heavy metal concentrations in sediment were high which could be attributed to nearby industries dumping their waste.

The changing pattern of elements in sediment samples can be due to geochemical processes but in living biomass it might be that roots store elements in large proportion with passing time and leaves use elements according to their requirements [41]. 

### 3.1. Stable Isotopes Signatures

#### 3.1.1. Leaves

The δ13C values ranged from −27.90 to −30.67‰ in all six compartments. Scientists mostly rely on the leaf chemistry of trees for δ13C measurements since it gives more information about forest dynamics. A value of δ13C indicates water use efficiency, salinity, and in some cases eutrophication [7]. These values showed less salinity stress for all six compartments. They also showed freshwater inputs for these mangrove compartments. A Santa Catarina, Brazil Tognella et al. [42] study showed values of −12.7 to −26.4‰, representing salinity stress in that area. 

Values of δ15N from 3.57 to 8.67‰ in leaves were positive, showing that N was not restricting plant development and that plants were acquiring their N from soil layers where denitrification represents a relative gathering of 15N [43]. Fry and Cormier, [7] explained δ15N values with watershed inputs. These values in leaves explain the anthropogenic effect of water irrigation in the form of Nitrogen pollution. In short, mangrove coastal ecosystems can be assessed for environmental stresses, sewage, and eutrophication by monitoring leaf nutrient status. 

**Figure 7:**
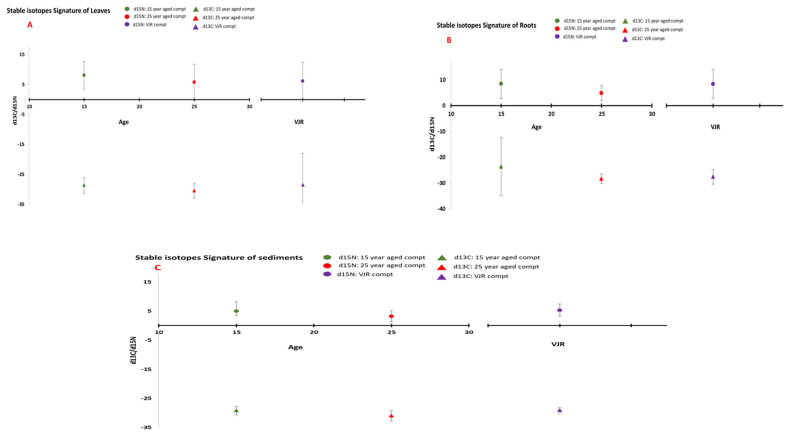
Stable Isotopes Concentration in (**A**) (leaves), (**B**) (roots), and (**C**) (sediments) in three age classes (mean was taken for same age compartments).

Stable isotope differences for the three age classes were also compared. In leaf samples, it was observed that there was no significant difference between different age classes (Figure 7), which reflects that studied species respond well under salinity and environmental stresses [44].

#### 3.1.2. Roots

Mangrove roots are actively involved in nutrient and water absorption. This prop root system helps mangroves to survive in difficult environmental conditions [45]. Furthermore, a horizontal network of roots covers a large area, enabling it to acquire more nutrients from the soil. Approximately more than 50% of biomass is allocated to roots by the mangrove trees.

Values of δ13C varied between −20.641 to −28.409‰ in all six compartments. These increased values depicted that roots also act as a carbon sink and enrichment of roots with 13C may significantly differ from other parts of mangrove trees [46]. 

Due to the denitrification role of mangrove roots, it had a positive input of δ15N ranging from 4.473 to 9.863‰ in this study. Furthermore, roots that were closely intact with soil also affect the elemental concentration in relation to various environmental stresses.

Within the three age classes, a significant difference can be observed in isotopic ratios (Figure 7). It may be due to changes in humidity, temperature, salinity, or tidal inundation on the mangrove ecosystem with increasing age [47].

#### 3.1.3. Sediment

Carbon concentration ranged from −28.82 to −31.27‰ in all six compartments (Figure 7). This range was more than the expected range for wetlands [48]. This range could be more positive with sediment depth. Values of δ13C showed there was less saline stress on vegetation. These values also indicated less decomposition of organic matter in sediments. At Santa Catarina, Brazil [26], δ13C ranged from about −22 to −24.6‰, a value that was higher compared to our study. On the other hand, a study by Lacerda et al. [49] in Sepetiba Bay in Rio do Janeiro supports the outcomes of our study.

The N sediment isotope signatures were positive, ranging from 2.66 to 9.86‰ in all six compartments. This result illustrates that all six sites are categorized by an open nitrogen cycle [50] and denitrification increases the accumulation of 15N. Moreover, it provides evidence as these sites not being deficient in soil N, as is the case in Santa Catarina, Brazil mangrove plantation, but we cannot solely rely on δ15N for N abundance [7].

### 3.2. Sampling Site

**Figure 8:**
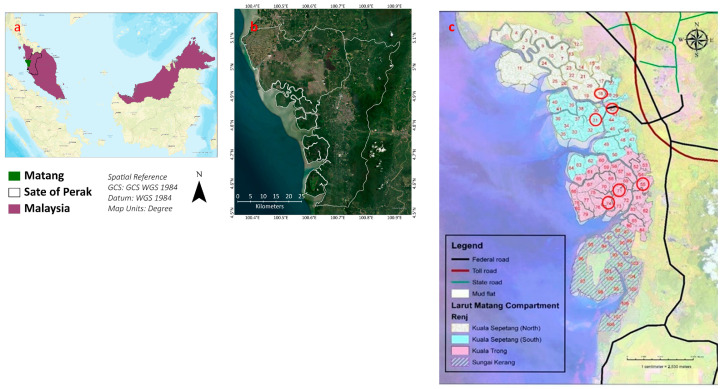
(**a**) A map of Malaysia with the Matang area and Perak state, (**b**) the MMFR and state of Perak, and (**c**) encircled sampling points.

The samples were collected from the Matang mangrove forest reserve. It is situated in Peninsular Malaysia at Perak. The total area of the MMFR is approximately 40,466 ha, and it is divided into four portions, which include Sungai Kerang, Kuala Sepetang South, Kuala Trong, and Kuala Sepetang North. These portions are subdivided into compartments (Compt.) for managerial purposes. Most compartments are classified as productive forests planted with *Rhizophora apiculata* and *R. mucronata*. However, several compartments are left untouched and are totally protected areas known as Virgin Jungle Reserve (VJR). The compartments which are used for timber extraction and undergo a thinning process by management are called managed compartments. On the other hand, the VJR and untouched compartments for many years are called unmanaged compartments. Six compartments were selected, three from Kuala Sepetang namely Compt. 18, 31, and VJR (Compt. 42), and three other Compt. from Kuala Trong, i.e., Compt. 71, 74, and VJR (Compt. 55). Compartments 18 and 31 are 15 years old, while Compt. 71 and 74 are 25 years old. VJR of Compt. 42 (Kuala Sepetang) and 55 (Kuala Trong) are taken as a control to compare the elements with the corresponding compartments of their areas as shown in Figure 8. 

### 3.3. Sample Collection

Transects were made in each compartment from seaward to landward. Three sample plots of 10 m × 10 m were made on this transect with equal gaps of 20 m between them. Based on our observation and inventory record from the Forestry Department, the dominant species in all compartments is *Rhizophora apiculata* except for Compt. 42 and Compt. 55 in which both *R. apiculata* and *R. mucronata* are dominant species. Because of that, five mature trees of *R. apiculata* were sampled. In order to increase the significance of the analysis, three replicates were made that resulted in a total of 270 samples (Table 4). Tree leaves were collected from those six compartments as explained in the study site above. For roots, samples of approximately 2 cm in diameter were collected at 7 cm depth. About 300 g of sediment samples were taken close to the sampled tree at about 7 cm depth from the sediment surface. All samples were placed in an ice box (4 °C) to avoid contamination and sample degradation after packing in zip-lock plastic bags individually [17,51].

### 3.4. Samples Preparation and Chemical Analysis

All collected samples were taken and processed in the soil laboratory at the Faculty of Forestry Universiti Putra Malaysia (UPM). Leaves and roots were rinsed with deionized water and all samples including sediment were dried at 60 °C to achieve a constant weight. Dried samples were ground by pestle and mortar and sieved using a 53 μm sieve. The digestion method was used for the homogeneity of ground samples by Zulkifli et al. [52] and Khan et al. [1]. In the digestion method, samples were briefly put in a digestion tube with concentrated nitric acid (HNO_3_) and were heated on a digestion block for 1 h and followed by cooling at room temperature. Cooled samples were filtered using Whatman filter paper into a plastic container and diluted with distilled water to achieve a fixed volume. Elements Mg, P, Cd, Cu, Fe, K, Mn, Zn, Ca, Na, and Pb were directly analyzed using Shimadzu Flame Atomic Absorption Spectrometer [(FAAS), model: AA-700, Japan] in the soil lab. The reference material is GSS-1 for sediments and GSV-2 for leaves. Phosphorus percentage (P %) was analyzed through the blue method [53] for all the samples. For finding the CNS percentage, a Trumac CNS Analyzer was used in the Faculty of Agriculture, UPM.

### 3.5. Stable Isotopes (13C and 15N) Samples Preparation and Analysis

Leaf and root samples were cleaned, washed, and dried at 60 °C to achieve a fixed weight. Dried samples were then ground to make a fine powder. For the removal of inorganic carbonates, the powdered samples were fumed with 12M HCl (analytical reagent (assay ≥ 37%); Sigma-Aldrich; USA) for at least 10 h [54]. For stable isotopes (13C and 15N) analysis, the samples were analyzed at the Malaysian Nuclear Agency, Bangi, Malaysia. A continuous flow isotopic ratio mass spectrometer with an elemental analyzer (CF-IRMS-EA) instrument was used to acquire stable carbon and nitrogen (13C/12C, 15N/14N) ratios. The stable carbon and nitrogen isotope ratios are expressed in delta (δ) notation (13C and δ15N) in units of parts per thousand (‰), as represented by the following equation:

δX = [(Rsample/RStandard) − 1] × 10^3^

where X is 13C and 15N, and R is the corresponding ratio.

### 3.6. Data Analysis

The elemental composition of leaves, roots, and sediment was analyzed with SPSS version 25. Stable isotopes and elemental differences between compartments were analyzed through One-way ANOVA. Age-based compartment comparison of stable isotopes and elemental concentration were also analyzed through One-way ANOVA. To assess homogeneity and non-homogeneity within samples, Tukey Hsd and Dunnett tests were performed to compare the nutrients in all compartments. Tree parts and sediment samples of all the Compartments were differentiated into groups based on significant differences as shown in Table 1, Table 2 and Table 3. 

## 4. Conclusions

Based on our study, compartment comparison gives the following average elemental concentration trend: Cd_(S)_ < Pb_(S)_ < Cu_(S)_ < Zn_(S)_ < Mg_(S)_ < Mn_(L)_ < K_(L)_ < Fe_(S)_ < Na_(R)_ < Ca_(R)_ and P%_(S)_ < S%_(S)_ < N%_(L)_ < C%_(R)_ in leaf, root and sediment samples. Leaves, roots, and sediment analysis for six compartments showed similar average elemental concentrations. The difference in elemental concentration is attributed to age or location. The mangrove forest regulates elements better than other forests. Sediments and roots are the main storage spaces for elements in the sampled mangrove area hence depicting that above-ground biomass is not a good sink for elemental storage. Sediment results however displayed an increase in elemental composition over time as compared to roots and leaves. Many elements in the three classes exhibited significant differences within the three age groups. These mangrove ecosystems have a high potential for carbon sequestration in sediments. Concentrations of Cu, Cd, Pb, Zn, Mn, Fe, P, and S were very high, particularly in sediment samples, which could be due to heavy metal pollution. The sources of these heavy metals and other elements such as N, P, and S, among others, can be from industries and agricultural activities in the catchment of the mangrove ecosystem under study. It is suggested to determine the risk assessment status of elemental concentration in sediments of the MMFR. This study provides baseline data for elements of different aged compartments, and it can be used for further research, comparison to different mangrove sites, and devising management policies. 

All the elements in mangrove leaves, except for Cu, C, and S, have an impact on compartment variation, and for *Rhizophora apiculata* species aged 15 from other compartments and ages. Elements Fe, K, Zn, Mg, and Na have an impact on the variations between compartments 18 and 31. For mangrove sediment, compartments 18, 42, 71, and 74 were influenced by Cd, P, N, C, and S based on the three mangrove age groups.

## Figures and Tables

**Table 1:** The chemical composition of *Rhizophora apiculata* leaves in six compartments (18, 31, 71, 74, 42, and 55).

		*Compartment 18* *n = 15*		*Compartment 31* *n = 15*		*Compartment 71* *n = 15*		*Compartment 74* *n = 15*		*Compartment 42* *n = 15*		*Compartment 55* *n = 15*	
Parameter	Units	Mean	95%CL	Mean	95%CL	Mean	95%CL	Mean	95%CL	Mean	95%CL	Mean	95%CL
δ15N	‰	7.52 ^a^	3.005	8.67 ^a^	2.06	7.92 ^a^	4.37	3.57 ^a^	1.23	5.03 ^a^	2.28	7.07 ^a^	3.71
δ13C	‰	−27.90 ^a^	1.9	−29.34 ^a^	0.78	−30.54 ^a^	1.97	−30.37 ^a^	0.36	−31.43 ^a^	6.17	−29.67 ^a^	2.22
Cd	mg/kg	0.0046 ^b^	0.0020	0.016 ^a^	0.002	0.0107 ^a^	0.003	0.0098 ^ab^	0.004	0.006 ^ab^	0.004	0.0135 ^a^	0.005
Cu	mg/kg	0.107 ^c^	0.007	0.052 ^abc^	0.016	0.067 ^ab^	0.025	0.117 ^bc^	0.013	0.085 ^bc^	0.024	0.076 ^a^	0.021
Fe	mg/kg	2.156 ^a^	1.036	7.116 ^a^	4.63	2.59 ^a^	1.18	1.505 ^a^	0.731	3.75 ^a^	1.88	2.145 ^a^	1.60
K	mg/kg	29.40 ^a^	2.33	31.04 ^a^	1.64	29.41 ^a^	1.61	27.17 ^a^	4.07	30.70 ^a^	1.76	29.81 ^a^	3.29
Mg	mg/kg	1.78 ^a^	0.13	1.85 ^a^	0.08	1.84 ^a^	0.08	1.65 ^a^	0.17	1.82 ^a^	0.1	1.82 ^a^	0.01
Mn	mg/kg	2.03 ^b^	0.088	3.14 ^ab^	1.17	3.57 ^ab^	1.26	2.30 ^b^	0.75	5.36 ^a^	2.07	4.14 ^a^	0.89
Pb	mg/kg	0.046 ^c^	0.18	0.11 ^ab^	0.037	0.143 ^a^	0.026	0.071 ^bc^	0.03	0.066 ^bc^	0.034	0.099 ^bc^	0.03
Zn	mg/kg	0.428 ^b^	0.127	0.757 ^a^	0.164	0.536 ^ab^	0.162	0.382 ^b^	0.126	0.573 ^ab^	0.239	0.445 ^b^	0.1
Na	mg/kg	4618.20 ^ab^	380.32	4806.66 ^a^	470.48	3881.06 ^b^	400.32	4414.06 ^ab^	542.94	4520.06 ^ab^	568.8	4946.80 ^a^	385.13
Ca	mg/kg	11,237.73 ^ab^	309.47	10,788.63 ^b^	269.91	10,996.73 ^ab^	364.67	10,808.86 ^ab^	435.65	11,378.40 ^a^	195.72	11,362.26 ^ab^	393.5
C	%	41.74 ^a^	1.51	39.096 ^ab^	1.19	34.25 ^b^	4.6	38.80 ^b^	0.42	39.98 ^b^	1.65	39.007 ^b^	0.5
N	%	0.970 ^a^	0.213	12.01 ^a^	11.42	1.67 ^a^	0.27	1.21 ^a^	0.19	1.55 ^a^	0.28	1.26 ^a^	0.367
P	%	0.222 ^b^	0.02	0.276 ^a^	0.07	0.246 ^a^	0.03	0.328 ^a^	0.05	0.290 ^a^	0.03	0.283 ^ab^	0.06
S	%	0.287 ^b^	0.02	0.367 ^ab^	0.04	0.419 ^ab^	0.04	0.357 ^a^	0.02	0.402 ^a^	0.02	0.344 ^ab^	0.06

Superscript letters (a, b, c) in each row indicate significance groupings. *p* < 0.05. Averages sharing the same superscript are similar and not significantly different and averages with different superscripts in each row are significantly different. See compartments 18 and 31 (15-year-old), 71 and 74 (25-year-old), and 42 and 55 (VJR).

**Table 2:** The chemical composition of *Rhizophora apiculata* roots in six compartments (18, 31, 71, 74, 42, and 55).

		*Compartment 18* *n = 15*		*Compartment 31* *n = 15*		*Compartment 71* *n = 15*		*Compartment 74* *n = 15*		*Compartment 42* *n = 15*		*Compartment 55* *n = 15*	
Parameter	Units	Mean	95%CL	Mean	95%CL	Mean	95%CL	Mean	95%CL	Mean	95%CL	Mean	95%CL
δ15N	‰	7.314 ^ab^	3.44	9.486 ^a^	2.688	4.473 ^b^	1.794	5.158 ^ab^	1.388	6.862 ^ab^	2.466	9.863 ^a^	3.437
δ13C	‰	−26.598 ^b^	2.635	−20.641 ^a^	8.178	−28.292 ^b^	1.132	−28.317 ^b^	0.8	−28.409 ^b^	1.3	−26.696 ^b^	1.6
Cd	mg/kg	0.0088 ^a^	0.004	0.0065 ^a^	0.002	0.0097 ^a^	0.002	0.0095 ^a^	0.005	0.0094 ^a^	0.002	0.007 ^a^	0.003
Cu	mg/kg	0.0994 ^a^	0.0413	0.0682 ^a^	0.02	0.0672 ^a^	0.02	0.0602 ^a^	0.015	0.106 ^a^	0.08	0.115 ^a^	0.08
Fe	mg/kg	5.985 ^a^	2.9	5.381 ^a^	3.06	5.608 ^a^	3.09	4.910 ^a^	2.51	6.656 ^a^	4.64	7.693 ^a^	3.836
K	mg/kg	28.074 ^a^	3.079	26.987 ^a^	3.44	26.552 ^a^	2.76	27.065 ^a^	3.63	23.928 ^a^	3.51	24.07 ^a^	3.44
Mg	mg/kg	1.833 ^a^	0.1	1.794 ^a^	0.18	1.832 ^a^	0.1	1.763 ^a^	0.13	1.764 ^a^	0.22	1.760 ^a^	0.17
Mn	mg/kg	0.834 ^a^	0.19	1.051 ^a^	0.23	0.582 ^a^	0.24	0.587 ^a^	0.15	2.069 ^a^	0.65	0.750 ^a^	0.27
Pb	mg/kg	0.0879 ^a^	0.034	0.0694 ^a^	0.036	0.0677 ^a^	0.033	0.0850 ^a^	0.032	0.0820 ^a^	0.031	0.0854 ^a^	0.0251
Zn	mg/kg	0.442 ^a^	0.125	0.513 ^a^	0.207	0.488 ^a^	0.154	0.412 ^a^	0.146	0.526 ^a^	0.237	0.470 ^a^	0.178
Na	mg/kg	5238.46 ^a^	309.06	5125 ^a^	431.2	5330.6 ^b^	252.5	5105.06 ^a^	118.96	5372.93 ^a^	241.4	5798.60 ^a^	453.98
Ca	mg/kg	10,843 ^b^	385.12	10,929.26 ^b^	401.26	9579.26 ^b^	1676.38	10,765.68 ^b^	466.07	10,992.86 ^b^	443.11	11,329.40 ^a^	467.35
C	%	39.534 ^a^	1.09	40.094 ^a^	1.20	39.660 ^a^	0.67	39.405 ^a^	1.05	38.452 ^b^	1.03	37.0307 ^b^	0.85
N	%	0.463 ^ab^	0.04	0.392 ^b^	0.03	0.551 ^a^	0.07	0.542 ^a^	0.05	0.507 ^a^	0.047	0.547 ^a^	0.058
P	%	0.262 ^a^	0.03	0.253 ^a^	0.03	0.224 ^a^	0.016	0.244 ^a^	0.028	0.268 ^a^	0.085	0.200 ^a^	0.014
S	%	0.211 ^b^	0.013	0.194 ^b^	0.009	0.246 ^a^	0.019	0.266 ^a^	0.033	0.535 ^a^	0.409	0.277 ^a^	0.051

Superscript letters (a, b) in each row indicate significance groupings. *p* < 0.05. See compartments 18 and 31 (15-year-old), 71 and 74 (25-year-old), and 42 and 55 (VJR).

**Table 3:** The chemical composition of sediments from six compartments in 18, 31, 42, 71, 74, and 55.

		*Compartment 18* *n = 15*		*Compartment 31* *n = 15*		*Compartment 71* *n = 15*		*Compartment 74* *n = 15*		*Compartment 42* *n = 15*		*Compartment 55* *n = 15*	
Parameter	Units	Mean	95%CL	Mean	95%CL	Mean	95%CL	Mean	95%CL	Mean	95%CL	Mean	95%CL
δ15N	‰	3.219 ^b^	1.22	6.675 ^a^	1.85	2.664 ^b^	1.4	3.649 ^b^	0.68	3.933 ^b^	0.9	6.556 ^a^	1.07
δ13C	‰	−29.272 ^a^	0.9	−29.176 ^a^	0.72	−30.738 ^b^	0.71	−31.272 ^b^	1.2	−28.825 ^a^	0.65	−29.307 ^a^	0.33
Cd	mg/kg	0.017 ^a^	0.009	0.12 ^a^	0.11	0.015 ^a^	0.005	0.016 ^a^	0.006	0.0113 ^a^	0.005	0.112 ^a^	0.102
Cu	mg/kg	0.068 ^ab^	0.02	0.098 ^ab^	0.028	0.052 ^bc^	0.017	0.55 ^bc^	0.51	0.135 ^a^	0.049	0.041 ^c^	0.01
Fe	mg/kg	40.552 ^a^	14.43	39.232 ^a^	12.92	39.329 ^a^	13.68	42.152 ^a^	16.27	46.321 ^a^	13.53	36.253 ^a^	11.97
K	mg/kg	28.930 ^a^	1.77	24.351 ^bc^	2.23	27.958 ^ab^	1.37	28.119 ^a^	1.78	28.016 ^ab^	1.14	22.088 ^a^	2.91
Mg	mg/kg	1.65 ^a^	0.13	1.94 ^a^	0.07	1.923 ^a^	0.11	1.967 ^a^	0.08	1.942 ^a^	0.09	1.928 ^a^	0.09
Mn	mg/kg	1.824 ^a^	0.3	1.126 ^ab^	0.4	1.514 ^ab^	1.01	2.032 ^a^	0.62	2.163 ^a^	0.73	0.882 ^b^	0.196
Pb	mg/kg	0.244 ^a^	0.056	0.283 ^a^	0.06	0.204 ^a^	0.056	0.236 ^a^	0.06	0.268 ^a^	0.039	0.185 ^a^	0.053
Zn	mg/kg	0.727 ^ab^	0.186	0.722 ^ab^	0.15	0.629 ^b^	0.15	0.703 ^ab^	0.155	0.998 ^a^	0.16	0.500 ^b^	0.05
Na	mg/kg	5156.68 ^a^	172.78	5069.06 ^a^	234.3	5084.06 ^a^	167.82	5122.46 ^a^	110.44	5099.93 ^a^	222.69	4851.66 ^a^	261.44
Ca	mg/kg	10,812.26 ^a^	186.59	10,624.86 ^a^	213.14	10,793.40 ^a^	353.3	10799 ^a^	305.13	9448.2 ^b^	1599.9	10,817.86 ^a^	348
C	%	9.425 ^abc^	2.59	8.876 ^bc^	0.861	10.842 ^a^	0.74	9.657 ^ab^	0.36	9.565 ^ab^	1.27	7.278 ^c^	0.7
N	%	0.380 ^bc^	0.066	0.437 ^ab^	0.034	0.477 ^a^	0.027	0.415 ^a^	0.02	0.386 ^bc^	0.031	0.319 ^c^	0.044
P	%	0.376 ^a^	0.028	0.201 ^b^	0.009	0.281 ^b^	0.05	0.380 ^a^	0.05	0.337 ^ab^	0.05	0.261 ^b^	0.05
S	%	0.760 ^bc^	0.323	0.542 ^c^	0.06	1.156 ^b^	0.188	1.262 ^ab^	0.18	1.705 ^a^	0.26	0.610 ^c^	0.05

Superscript letters (a, b, c) in each row indicate significance groupings. *p* < 0.05. See compartments 18 and 31 (15-year-old), 71 and 74 (25-year-old), and 42 and 55 (VJR).

**Table 4:** Number of samples collected with the environmental parameter information of each compartment together.

Compartments	Leaves Samples No.	Root Samples No.	Soil Samples No.	Compartment Age	Water Salinity (ppt)	Water Ph	River Name	Species	Status	DBH (cm)	Density (ha^−1^)	Phytomass (T ha^−1^)
18	15	15	15	15 years	20.3	4.6	Crying river	*R. apiculata*	Unmanaged	5–15	2075	235
31	15	15	15	15 years	18.9	5	Sanga besar	*R. apiculata*	Unmanaged	6–15	1901	168
42	15	15	15	VJR	20.7	4.9	Crying river	*R. apiculata*	Managed	4–15	1084	125
71	15	15	15	25 years	18.8	6.2	Mongokok	*R. apiculata*	Unmanaged	7–35	1287	241
74	15	15	15	25 years	23.2	4.5	Sungai tiram dilam	*R. apiculata*	Unmanaged	6–36	1175	283
55	15	15	15	VJR	19.8	4.8	Sungai trong	*R. apiculata*	Managed	6–35.3	1690	266

Total number of samples = 270, VJR = Virgin Jungle Reserve, DBH = Diameter at breast height.

## Data Availability

Not applicable.
